# P50 Antibiotics at a glance: driving stewardship through live dashboards

**DOI:** 10.1093/jacamr/dlag102.056

**Published:** 2026-06-26

**Authors:** Fiona Lynch, Anna Tilley, Natalie Jones, Kathryn Ashton, Nicola Robinson, Katie Wainwright, Christopher Dixon

**Affiliations:** Manchester University NHS Foundation Trust, Manchester, UK; Manchester University NHS Foundation Trust, Manchester, UK; Manchester University NHS Foundation Trust, Manchester, UK; Manchester University NHS Foundation Trust, Manchester, UK; Manchester University NHS Foundation Trust, Manchester, UK; Manchester University NHS Foundation Trust, Manchester, UK; Manchester University NHS Foundation Trust, Manchester, UK

## Abstract

**Background:**

Timely review of antimicrobial therapy is a core principle of antimicrobial stewardship (AMS), yet ward teams often lack rapid access to actionable prescribing information. Delays in reviewing IV therapy, broad-spectrum agents, or time-dependent prescriptions can contribute to avoidable antimicrobial exposure, suboptimal patient flow, and increased risk of healthcare-associated infections (HCAIs).

**Objectives:**

To develop and implement a live AMS dashboard within the electronic prescribing and medicines administration (EPMA) system, providing ward teams with real-time visibility of patients receiving antimicrobials. The dashboard was designed to prompt and prioritize clinical review, supporting safe, timely optimization of therapy.

**Methods:**

A multidisciplinary team created an integrated dashboard displaying key stewardship metrics drawn directly from prescribing data. Metrics included: all patients currently receiving IV antimicrobials for 72 h or longer; antimicrobials due to expire within 24 h; use of broad-spectrum antibiotics; agents requiring therapeutic drug monitoring (TDM); and IV antimicrobials with high oral bioavailability suitable for IV-to-oral switch. The dashboard can be filtered by hospital, clinical specialty or ward area with the aim to embed use in ward and board rounds to promote timely review of antimicrobials.

**Drivers:**

The intervention targeted improvements in patient flow, reduction in unnecessary broad-spectrum and IV antimicrobial use, and decreased incidence of HCAIs through earlier optimization of therapy.

**Results and conclusions:**

Implementation of a live AMS dashboard has the potential to enhance situational awareness, streamline antimicrobial review processes, and support stewardship-aligned decision-making at the point of care. Early findings suggest improved identification of patients for targeted AMS ward round, intermittent use in some ward areas during ward rounds, board rounds and safety huddles. Knowledge and promotion of the dashboard across the organization remains a challenge and further promotional work is required to optimize its utility.

